# Novel Self-Nano-Emulsifying Drug Delivery Systems Containing Astaxanthin for Topical Skin Delivery

**DOI:** 10.3390/pharmaceutics13050649

**Published:** 2021-05-03

**Authors:** Thellie Ponto, Gemma Latter, Giuseppe Luna, Vânia R. Leite-Silva, Anthony Wright, Heather A. E. Benson

**Affiliations:** 1 Curtin Medical School, Curtin Health Innovation Research Institute (CHIRI), Curtin University, GPO Box U1987, Perth, WA 6845, Australia; thellie.ponto@postgrad.curtin.edu.au (T.P.); gemma.latter@postgrad.curtin.edu.au (G.L.); giuseppe.luna@curtin.edu.au (G.L.); 2 Instituto de Ciências Ambientais, Químicas e Farmacêuticas, Departamento de Ciências Farmacêuticas, Universidade Federal de São Paulo, UNIFESP-Diadema, São Paulo 09913-030, Brazil; vaniarleite@uol.com.br; 3 School of Allied Health, Curtin University, GPO Box U1987, Perth, WA 6845, Australia; t.wright@curtin.edu.au

**Keywords:** skin targeting, antioxidant, terpenes, penetration enhancement, SNEDDS, nano-delivery, cosmeceutical, dermatological

## Abstract

Astaxanthin (ASX) is a potent lipophilic antioxidant derived from the natural pigment that gives marine animals their distinctive red-orange colour and confers protection from ultraviolet radiation. Self nano-emulsifying drug delivery systems (SNEDDS) have been successfully developed and evaluated to increase the skin penetration of ASX and target its antioxidant and anti-inflammatory potential to the epidermis and dermis. SNEDDS were prepared using a low-temperature spontaneous emulsification method, and their physical characteristics, stability, antioxidant activity, and skin penetration were characterized. Terpenes (D-limonene, geraniol, and farnesol) were included in the SNEDDS formulations to evaluate their potential skin penetration enhancement. An HPLC assay was developed that allowed ASX recovery from skin tissues and quantification. All SNEDDS formulations had droplets in the 20 nm range, with low polydispersity. ASX stability over 28 days storage in light and dark conditions was improved and antioxidant activity was high. SNEDDS-L1 (no terpene) gave significantly increased ASX penetration to the stratum corneum (SC) and the epidermis-dermis-follicle region (E + D + F) compared to an ASX in oil solution and a commercial ASX facial serum product. The SNEDDS-containing D-limonene gave the highest ASX permeation enhancement, with 3.34- and 3.79-fold the amount in the SC and E + D + F, respectively, compared to a similar applied dose of ASX in oil. We concluded that SNEDDS provide an effective formulation strategy for enhanced skin penetration of a highly lipophilic molecule, and when applied to ASX, have the potential to provide topical formulations for UV protection, anti-aging, and inflammatory conditions of the skin.

## 1. Introduction

**Figure 1:**
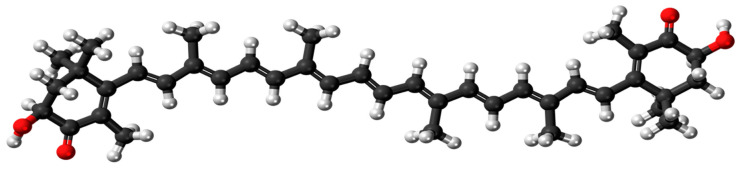
Structure of all-*trans* astaxanthin (C_40_H_52_0_4_: carbon—black, hydrogen—white, oxygen—red). By Jynto: this image was created with Discovery Studio Visualizer, CC0, https://commons.wikimedia.org/w/index.php?curid=16311511 (accessed on 9 February 2021).

There is increasing consumer demand for effective, natural-based products to protect the skin from environmental assault and treat dermatological conditions related to skin aging, irritancy, and inflammation [1]. Astaxanthin (3,3′-dihydroxy-β,β-carotene-4,4′-dione; ASX: Figure 1) is a carotenoid from the xanthophylls family with potent antioxidant activity [2]. ASX is commonly found in nature and is best known as the red-orange pigment that contributes the distinctive colour to many marine animals such as salmon, shrimp, and crayfish, and the flamingo birds that feast on them. It has many important biological functions in marine animals including pigmentation, protection against ultraviolet (UV) light effects, communication, immune response, reproductive capacity, stress tolerance, and protection against oxidation of macromolecules [3]. This keto-carotenoid is synthesized by a variety of green microalgae/phytoplankton, and also red yeast, bacteria, and other plants [4].

ASX has attracted interest due to its reported therapeutic effects in a range of health conditions [5]. This includes the prevention and treatment of cardiovascular diseases [2,6,7,8], neurological diseases [9,10,11], ocular disorders [12,13], cancer [14] and improving dermal health [15,16]. ASX derived from the green microalga *Haematococcus pluvialis* is the main source for human applications including dietary supplements, cosmetics, and food. It has shown antiaging potential in an experimental model of oxidative stress [17]. This suggests its potential in protecting human skin from environmental-derived stress, such as cigarette smoking and ultraviolet (UV) exposure, that contributes to and accelerates the skin-aging effects.

Skin aging has both cosmetic and health implications. The molecular and morphological changes that occur as the skin ages reduce its barrier function and protective role, and contribute to a range of skin symptoms including the formation or deepening of wrinkles, dyspigmentation, excessive dryness and pruritus, fragility, and difficulty in healing following skin injuries, skin sensitivity and irritation, and tumor incidence [15]. A range of positive effects on human skin has been reported following dietary supplementation with ASX [18,19,20,21,22,23]. In particular, ASX has shown to suppress hyperpigmentation and melanin synthesis, improve skin elasticity, inhibit photoaging associated with UV light exposure, and reduce wrinkle formation [18,19,20,21,22,23]. For example, in a double-blind, placebo-controlled trial, Ito et al. [18] showed that dietary supplementation with ASX for 9 weeks reduced the UV-induced changes in skin moisture and texture in 23 healthy volunteers. In another study conducted in Japan on 65 healthy female participants, those in the placebo group showed worsening wrinkle parameters, skin moisture decrease, and increase in the inflammatory marker interleukin-1α over a 16 week period from August to December [22]. Those receiving oral ASX showed no change in these skin parameters. The authors concluded that oral ASX supplementation may inhibit age-related skin deterioration via its anti-inflammatory effect. In most cases, these studies involve dietary supplementation rather than topical administration of ASX directly to the skin.

ASX (MW: 596.84 g/mol; Figure 1) has a high log P (octanol/water) of 13.27 [24], exhibiting low water solubility and limited bioavailability [25]. When applied to the skin, the ASX may not be efficiently released from an oil-based formulation, or may form a reservoir in the lipid-rich regions of the stratum corneum and not permeate further into the viable skin tissues, thus limiting its accessibility to its site of therapeutic activity. ASX is highly sensitive to light, heat, and oxygen due to its unsaturated molecular form [26]. Consequently, ASX presents significant challenges for the development of a successful formulation that will optimize stability and bioavailability within the skin. Commercially available topical formulations of ASX are primarily oil-based serums such as astaxanthin in argan oil (Skin Actives Scientific L.L.C., Gilvert, AZ, USA), Astalift Essence Destiny (FujiFilm Healthcare Laboratory Co., Ltd., Minato-ku, Tokyo, Japan), and Astarism (AstaREAL Co., Ltd., Minato-ku, Tokyo, Japan). A number of novel delivery systems have been investigated to facilitate the formulation of ASX, improve stability and skin permeation. These include micro/nanoemulsions [27,28], hydrogels/lipogels [29], liposomes [30], and nanostructured lipid carriers (NLCs) [31]. Whilst some of these approaches appear promising, there remains limited information based on robust in vitro permeation tests (IVPT) with appropriate IVPT protocols and skin membranes, to properly assess ASX delivery into skin target sites where it can exert its therapeutic activity. For example, in some studies, IVPT was conducted using rat [27,28,31] or mouse skin [30], which are widely acknowledged to be more permeable than human skin; and in one case, only an in vitro release test (IVRT) was performed [29].

In the current study, we have formulated self-nano-emulsifying drug delivery systems (SNEDDS) and evaluated the inclusion of terpene components on the skin permeation, stability, and antioxidant activity of ASX. SNEDDS are isotropic mixtures of an active compound in a combination of lipids, surfactants, and hydrophilic co-solvents/solubilizers that produce spontaneous ultrafine emulsions upon gentle agitation in the water phase (typically <50 nm in size) [32,33,34]. SNEDDS have a high solubilizing capacity for lipophilic compounds, are thermodynamically stable, provide simplicity of fabrication, and have an elegant appearance [35,36]. They have been shown to enhance skin delivery of lipophilic solutes such as curcumin [37] and clofazimine [38]. When applied topically, a SNEDDS may be diluted by an aqueous phase coming to the skin surface from the secretion of sweat or trans epidermal water loss, and then form an occlusive topical system that has a high thermodynamic driving force for skin delivery [34,39,40]. We suggest that the SNEDDS approach is ideally suited to deliver the highly lipophilic ASX into the skin to target sites of therapeutic action such as melanocytes located in the basal layer of the epidermis, collagen and elastin synthesis by fibroblasts located in the dermis and UV induced inflammatory processes in the epidermis and dermis [19,23,30]. SNEDDS also has potential advantages for ASX stability, as the elimination of water during preparation and storage reduces the potential for dissolved oxygen in the water phase of an emulsion to degrade ASX [28].

We have developed and characterized the physical characteristics, stability, skin permeation, and distribution, and antioxidant activity of SNEDDS composed of oil, non-ionic surfactants, and cosurfactants and incorporating ASX. We have also evaluated the inclusion of terpenes as potential skin penetration enhancers. To facilitate this study, we have developed a simple and accurate HPLC assay for ASX determination with suitable extraction protocols for quantifying ASX in skin layers.

## 2. Material and Methods

### 2.1. Chemicals and Materials

Astaxanthin of analytical grade (CAS# 472-61-7) (SML0982) with a purity of 99.7%, Kolliphor^®^ EL (Polyoxyl 35 castor oil–CAS# 61791-12-6), D-limonene (CAS# 5989-27-5), geraniol (CAS# 106-24-1), farnesol (CAS# 4602-84-0), L-ascorbic acid (CAS# 50-81-7), potassium persulfate (CAS# 7727-21-1), and ABTS (Roche-Diagnostics GmbH) (CAS# 30931-67-0) were purchased from Sigma-Aldrich (North Ryde, Australia). Labrafil^®^ M 1944 CS (Oleoyl polyoxyl-6 glycerides–CAS# 69071-70-1) and Transcutol^®^ P (Ethoxydiglycol–CAS# 111-90-0) were kind gifts from Gattefossé (Saint-Priest, France). Astarism^®^ facial serum was a gift from AstaREAL Co. Ltd., Minato-ku, Tokyo, Japan. Veet^™^ hair removal cream (containing potassium thioglycolate) was purchased from Reckitt Benckiser Pty Ltd. (Sydney, Australia). HPLC-grade methanol and ethanol were from Thermo Fisher Scientific (Scoresby, Australia). HPLC-grade solvents (including acetone (ACT) and dichloromethane (DCM) were purchased from RCI Labscan Ltd. (Pathumwan, Bangkok, Thailand) distributed by Chem-Supply Pty Ltd., Gillman, Australia. Potassium chloride and sodium chloride were purchased from Chem-Supply Australia Pty Ltd. Potassium dihydrogen orthophosphate was purchased from Thermo Fisher Scientific (Scoresby, Australia). Di-sodium hydrogen orthophosphate anhydrous was purchased from Merck Pty Ltd (Bayswater, Australia). Deionised water was produced using a Milli-Q RC apparatus (Millipore Corporation, Bedford, MA, USA).

### 2.2. Standard Solution Preparation

A stock standard solution of astaxanthin was prepared by weighing exactly 3.75 mg of astaxanthin into a 25 mL volumetric flask, dissolving the astaxanthin in a mixture (15 mL) of acetone and dichloromethane (50:50), and adding a mobile-phase solution to 25 mL. The solution was sonicated for 1 minute in a warm water bath at 60 °C, and allowed to equilibrate to ambient temperature for 15 min. Working solutions of individual standards were prepared by diluting the freshly prepared stock solution with the mobile phase to required concentration. 

### 2.3. HPLC Instrumentation and Conditions

Chromatographic separation was performed using an Agilent^™^ 1200 system (Agilent Technologies, Waldbronn, Germany), using a Jupiter C18 5 µm column, (150 mm × 4.6 mm) protected by a Security Guard Cartridge (C18, 4 × 3 mm) both from Phenomenex (Lane Cove, Australia), and isocratic flow of mobile phase (methanol:water:dichloromethane = 85:13:2) at 1 mL/min (chromatography adapted from Yuan et al. [41]. Samples were maintained at 10 °C within the autoinjector. Full assay conditions and protocols for extraction from skin tissues are available in the Supplementary Materials.

### 2.4. Design of Self-Nanoemulsifying Drug Delivery Systems (SNEDDS) 

SNEDDS were formulated with Labrafil^®^ M 1944 CS (constituting oil phase) and Kolliphor^®^ EL (non-ionic surfactant), and Transcutol^®^ P as a co-surfactant. Three terpenes (D-limonene, geraniol, and farnesol) were added, as 5% *w*/*w* of the oil phase, to determine their ASX skin permeation enhancement capability within the SNEDDS formulation. The physical characteristics and ASX skin delivery of all formulations were characterized, including the droplet size, polydispersity index (PDI), viscosity, ASX solubility, stability, oxidative inhibition values, and amount of ASX retained in the skin (IVPT).

The ratio of Labrafil^®^ M 1944 CS, Kolliphor^®^ EL, and Transcutol^®^ P was determined from ASX solubility experiments. The SNEDDS were prepared based on the method of Hong et al. [27], using a spontaneous emulsification method, and high energy input. Briefly, ASX (3 mg) was dissolved in 4 g of surfactant, stirred with magnetic stirring at 1000 rpm for 10 min. The mixture was then dissolved in 1 g of the oil phase, with magnetic stirring at 1000 rpm for 10 min. This mixture was then combined with 1 g of co-surfactant using magnetic stirring for 10 min, then sonicated in a water bath for 1 h at room temperature. The formulation vehicle addition sequence was based on preliminary ASX solubility determination in each vehicle component. A nanoemulsion formulation (L1-NE) was made by the same method but with an initial 6 mg ASX and then a 50:50 dilution with water as the final step to provide a nanoemulsion with the same ASX concentration as the SNEDDS formulations.

The composition of the SNEDDS formulations is documented in Table 1 with the initial SNEDDS formulation designated L1 and the terpene-containing formulation designated T1 (D-limonene), T2 (geraniol), and T3 (farnesol). All ASX SNEDDS were protected from light exposure at room temperature for further use.

### 2.5. Physical Characterization, Stability, and Antioxidant Activity

The ASX SNEDDS were visually observed to describe the physical appearance (colour, clarity, single phase), and viscosity (Bohlin Visco 88, Malvern Instruments, Malvern, Worcestershire, UK), and refractive index (Atago refractometer, Minato-ku, Tokyo, Japan) were determined. The pH measurement was determined using a pH meter (Hanna Instruments, Woonsocket, RI, USA) for all SNEDDS anhydrous formulations and after 100-fold dilution with deionized water. The droplet size and polydispersity index (PDI) were examined using a Zetasizer Nano^™^ ZSP (Malvern Instruments, Malvern, Worcestershire, UK) with aqueous dilution 100-fold to avoid multiple scattering.

Robustness to dilution was assessed in different volumes (1 part to 50, 100, and 250) using deionized water. This parameter provides a prediction of the potential for phase separation of a system generated by spontaneous emulsification. After the dilution process, the droplet size should remain uniform [33].

The stability of ASX SNEDDS was determined over 4 weeks of storage at room temperature to provide a preliminary indication of the stability of ASX in the SNEDDS formulations, when exposed to or protected from light. Physical appearance and the ASX content were determined after storage at 22–25 °C in clear vials and protected from light in amber vials. ASX in oil solution was used as a control.

### 2.6. In Vitro Skin Penetration (IVPT) Study

Due to the lack of availability of human skin, newborn pig skin was used as a previously validated human skin surrogate [42,43]. Full-thickness skin was obtained from newborn Yorkshire piglets that had died at or within 24 h of birth, as supplied by a local veterinary service. The skin was peeled from the body and extraneous tissue was removed by surgical scalpel. Hair was removed using Veet™ hair removal cream applied for 10 min and then thoroughly removed. This method has been previously validated on piglet skin and shown no skin damage [43]. The skin surface was rinsed with phosphate-buffered saline solution at pH 7.4 (PBS), wrapped in aluminum foil, and stored in a polyethylene bag at −20 °C until use. Three different donors were used for each experiment (9 replications).

The skin was defrosted at ambient temperature immediately before each experiment. Skin thickness was determined by digital vernier caliper (Kincrome Australia Pty Ltd., Scoresby, Australia) and was 400–600 µm. The skin was mounted in Franz diffusion cells with the stratum corneum (SC) side facing the donor. PBS solution was placed in the donor and receptor compartments and allowed to equilibrate at 35 °C prior to determining the electrical resistance (UNI-T^®^, UT58 series), as a measure of skin barrier integrity. Skin was included that provided a maximum resistance of 1 MΩ and minimum value of 50 kΩ [44]. PBS was removed and the skin surface touched dry (Kimtech Science Kimwipes^®^, Kimberly-Clark Professional, Milsons Point, Australia). The receptor compartment was filled with a mixture of PBS and ethanol (7:3 *v/v*) to facilitate ASX solubility [28,29], stirred by magnetic stirring at 600 rpm/min, and maintained in a water bath system at 35 °C to provide skin surface temperature of 32 °C. Parafilm M^®^ (Sigma-Aldrich Pty Ltd, North Ryde, Australia) was used to cover the donor and receptor cells to minimize evaporation and to facilitate the process of total replacement sampling. 0.5 g of ASX SNEDDS formulation, marketed ASX product or ASX in oil solution (control) was applied to the donor compartment as an infinite dose. Samples (total receptor fluid volume) were withdrawn for HPLC analysis and immediately replaced by pre-warmed receptor solution, over an 8 h period.

#### Skin Distribution Study

After 8 h, the skin was washed to remove any remaining formulation (washing fluid retained), dried, and treated to separate SC and remaining skin tissue (epidermis/dermis). To obtain SC samples, D-Squame^®^ adhesive tapes with a diameter of 22 mm (CuDerm, Dallas, TX, USA) were applied on the skin surface with a D-Squame disc^®^ applicator following the method of Davies et al. [45] with modification. The total of 10 tapes strips comprised the first two tapes (assumed to include unabsorbed ASX) and remaining 8 tapes as absorbed into the SC. The remaining skin was cut into pieces and weighed. ASX was extracted by soaking in acetone: dicloromethane 50:50 mixture prior to sonication in a water bath for 1 h at room temperature. The supernatant fluid was collected and then centrifuged to remove the precipitation of fat tissue. The tapes were soaked in methanol, vortex mixed for 30 min, then removed and the soaking solution centrifuged for 10 min. All samples were analyzed as soon as possible by HPLC.

### 2.7. Antioxidant Activity

The antioxidant activity of ASX was determined by the ABTS (2,2′-azino-bis(3-ethylbenzothiazoline-6-sulfonic acid)) radical scavenging assay according to Chintong et al. [46] with minor modification. ABTS provides radicals that can be easily observed by a colour change from almost colourless to deep bluish-green in the absorbance wavelength at 734 nm [47].

Briefly, 7 mM ABTS and 2 mM K_2_S_2_O_8,_ both in aqueous solution and equal quantities, were allowed to stand in the dark at room temperature for 16 h. ABTS working solution was then diluted with distilled water to obtain an absorbance of 0.70 ± 0.03 units at 734 nm using a UV–Vis spectrophotometer (UV-1280, Shimadzu, Nakagayo-ku, Kyoto, Japan). Twenty microliters of different concentrations of the SNEDDS and ASX in ethanol 95% solution (200–1000 µg/mL) was mixed with 200 µL of the working ABTS solution. The absorbance of the mixture was measured after 6 min at wavelength 734 nm. Ascorbic acid and the marketed ASX topical product were compared as positive controls. Water and ethanol 95% were used as blanks. The scavenging rate of ABTS was calculated as:

%ABTS radical scavenging = [(A_sample_ − A_sample blank_)/A_control_] × 100

where A_control_, A_sample_, and A_sample blank_ are the absorbance of the ABTS solution, ASX solution with ABTS, and ASX solution without ABTS, respectively.

### 2.8. Data Analysis

For the in vitro skin permeation study, the amount of ASX in the skin tissues was calculated from the HPLC analysis of extract solutions giving the amount of ASX retained in the SC (tape strips 2–10) and the amount of ASX present in the skin tissue (epidermis, dermis).

Mass balance of in vitro permeation experiments was determined by comparison of the initial applied amount of ASX with the combination of ASX in the wash, tape strips 1/2 and 3–10 samples, the remaining skin tissue extracts, and the receptor compartment.

### 2.9. Statistical Analysis

All experiments were carried out in triplicate. The data are expressed as the mean ± SD (non-biologically related measurements) and the mean ± SEM (biologically related measurements). All data were analyzed using GraphPad Prism^™^ 9 software (GraphPad Software Inc., San Diego, CA, USA), using two-way ANOVA with Tukey post hoc test. Data were considered to be statistically significant if *p* < 0.05.

## 3. Results

### 3.1. HPLC Assay Method Development

The HPLC method provided a single ASX peak for the stock solution at 8.3 ± 0.1 min and good separation from any skin sample-related peaks (see Figure S1). The details of the assay development and outcomes are provided in the Supplementary Materials.

### 3.2. Preparation of ASX-Loaded SNEDDS

The initial SNEDDS formulation was developed based on solubility studies of ASX with the three core excipients of Labrafil^®^ M 1944 CS (constituting the oil phase), Kolliphor^®^ EL (non-ionic surfactant), and Transcutol^®^ P as a co-surfactant. The optimal ratio was 1:4:1 (oil:surfactant:co-surfactant) providing the SNEDDS-L1 formulation. This ratio provided a transparent SNEDDS formulation that readily dispersed in water to produce a nanoemulsion with suitably small droplet size. The SNEDDS-L1 formulation was then adapted to include a terpene in the oil phase, providing SNEDDS-T1 (D-limonene), T2 (geraniol), and T3 (farnesol). The addition of 5% D-limonene, geraniol, and farnesol generated transparent nanoformulations with excellent clarity and similar physical characteristics to the SNEDDS-L1 formulation.

### 3.3. Physical Characteristics of ASX Nanoformulations

**Figure 2:**
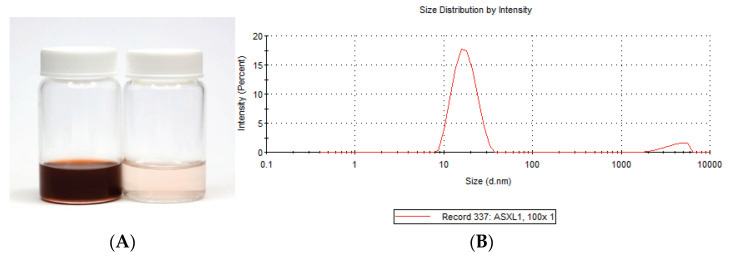
(**A**) Photographic image of SNEDDS–L1 (left) and SNEDDS–L1 diluted 100-fold by water (right). (**B**) Size distribution of SNEDDS–L1.

All SNEDDS formulations were a darkish red-orange colour that formed a pale orange transparent nanoemulsion system when diluted 100-fold by deionized water (Figure 2A). The colour is associated with the ASX. Addition of terpenes did not alter the physical appearance of the SNEDDS. There was no sedimentation or phase separation in any SNEDDS formulation when observed immediately after formulation or after up to 28 days storage at room temperature.

The droplet size and PDI were measured immediately after the preparation of each SNEDDS. In all cases the SNEDDS exhibited a monomodal distribution reflecting uniform size distribution, as shown for SNEDDS-L1 in Figure 2B. All SNEDDS formulations had a droplet size below 20 nm and with a polydispersity index of less than 0.25, indicating a narrow distribution pattern and homogeneity (Table 2). At ambient temperature, the SNEDDS were viscous and spread easily and smoothly on the skin surface. The viscosity ranged from 0.19 ± 0.01 Pas to 0.23 ± 0.01 Pas (Table 2). The pH for all SNEDDS was in the range of 6.17–8.01 and reduced to 4.43–5.42 after dilution, bringing them close to the pH range of the skin and similar to the marketed ASX product (Table 2).

The zeta potential of all SNEDDS formulations, determined after dilution with distilled water, were −13.10 ± 0.52 to −12.40 ± 0.20 mV, and results are summarised in Table 2. The relatively low value of zeta potential in the prepared self-nanoemulsifying formulations may be due to the presence of a relatively higher amount of Kolliphor^®^ EL used in formulations so that the surface charge was contributed only by co-surfactant.

The robustness to dilution test showed minimal change in the droplet size and PDI of the SNEDDS formulation upon dilution with 50-, 100-, and 250-fold deionised water with the droplet size remaining approximately 20 nm for all formulations (Table 3).

### 3.4. In Vitro Skin Penetration/Permeation Study 

No detectible ASX permeated through the skin to the receptor fluid from any of the SNEDDS, commercial formulation or ASX in oil (control) over the 8 h topical application period. In contrast, ASX was present in the SC and remaining skin comprised of the epidermis, dermis, and associated follicular regions (Table 4). 

**Figure 3:**
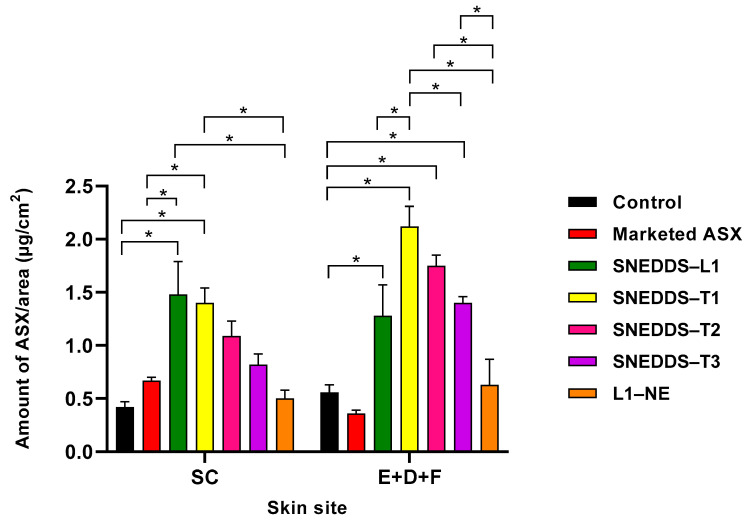
Skin penetration profile of SNEDDS formulations compared to marketed topical product and ASX in oil control: the distribution of ASX in the SC and E + D + F; (mean ± SEM; *n* = 9; * *p* < 0.05).

Figure 3 shows a comparison of the skin distribution of ASX following administration of the SNEDDS formulations, commercial topical product, and ASX in oil solution control. The SNEDDS–L1, comprised of Labrafil^®^ M 1944 CS, Kolliphor^®^ EL, Transcutol^®^ P, showed statistically significantly greater penetration of ASX into the SC compared to the marketed commercial product and control ASX in oil solution (1.48 ± 0.31; 0.67 ± 0.03; 0.42 ± 0.01 µg/cm^2^, respectively; *p* < 0.05). The SNEDDS also deposited statistically significantly more ASX in the deeper skin (E + D + F) than the commercial product and the oil solution control (1.28 ± 0.29; 0.36 ± 0.03; 0.56 ± 0.07 µg/cm^2^, respectively; *p* < 0.05). Overall, there was a 2.2-fold increase in ASX in the SC and a 3.6-fold increase in the amount of ASX in the (E + D + F) from SNEDDS–L1 compared to marketed ASX nanoformulation product.

### 3.5. Effect of D-Limonene, Geraniol, and Farnesol Incorporated into SNEDDS on Skin Penetration

Three terpenes were added to the oil phase of the SNEDDS-L1 formulation to create SNEDDS-T1 (D-limonene), SNEDDS-T2 (geraniol), and SNEDDS-T3 (farnesol), and their effect on skin penetration determined (Table 4; Figure 3). No ASX was detected in the receptor fluid after 8 h application of any of the SNEDDS-containing terpenes.

All SNEDDS-containing terpenes increased ASX permeation into the SC compared to ASX in oil solution control and marketed topical product. However, the ASX penetration was significantly greater only from the SNEDDS-T1 formulation. In comparison to the SNEDDS-L1, only the SNEDDS-T1 provided similar ASX penetration into the SC, with the SNEDDS-containing geraniol and farnesol showing lower but not significantly different ASX penetration. When considering ASX penetration into the deeper skin tissues (E + D + F), the terpene-containing SNEDDS all showed statistically significantly greater ASX penetration than the control and marketed topical product, with penetration in the rank order of SNEDDS-T1 > T2 > T3. In addition, all terpene-containing SNEDDS showed greater penetration than SNEDDS-L1, although the difference was significantly different only for the D-limonene formulation.

The SNEDDS-L1 formulation was converted to an aqueous bearing nanoemulsion (designated L1-NE) by the addition of 50% water whilst maintaining the ASX concentration of the final nanoemulsion formulation. Addition of water significantly reduced the ASX penetration to the SC and (E + D + F) in comparison to the SNEDDS-L1 formulation (Table 4). ASX penetration from the L1-NE was reduced compared to all SNEDDS formulations (Figure 3).

### 3.6. Preliminary Stability Study of ASX SNEDDS during Storage

**Figure 4:**
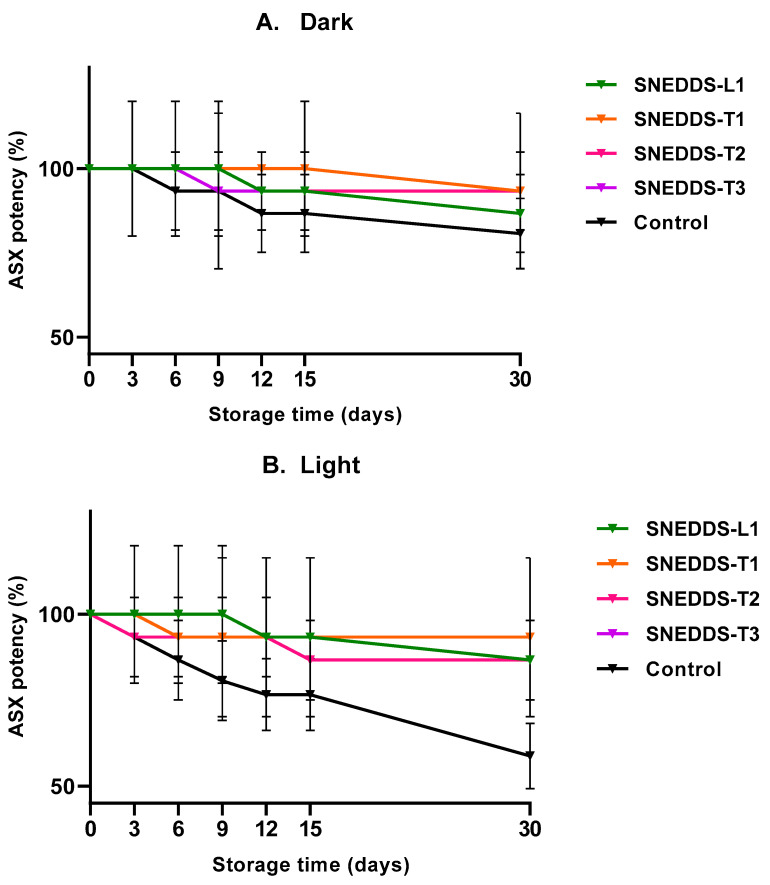
% ASX remaining after 30 day storage of SNEDDS formulations and ASX in oil (control) at room temperature in the dark (**A**) and light (**B**) conditions (mean ± SD; *n* = 3).

ASX stability was determined for all SNEDDS formulations and a control solution of ASX in oil, over 1 month storage at 22–25 °C with light-protected (dark) and unprotected light conditions. ASX% remaining after 30 days was in the range of 86.67–93.33% for both dark and light conditions for the SNEDDS formulations (Figure 4A,B). The ASX in oil solution was relatively stable for 30 days at 22–25 °C when in the dark (80.74 ± 11.55%) but was reduced significantly when exposed to the light (58.75 ± 9.55%).

### 3.7. Antioxidant Activity of ASX SNEDDS Formulations

**Figure 5:**
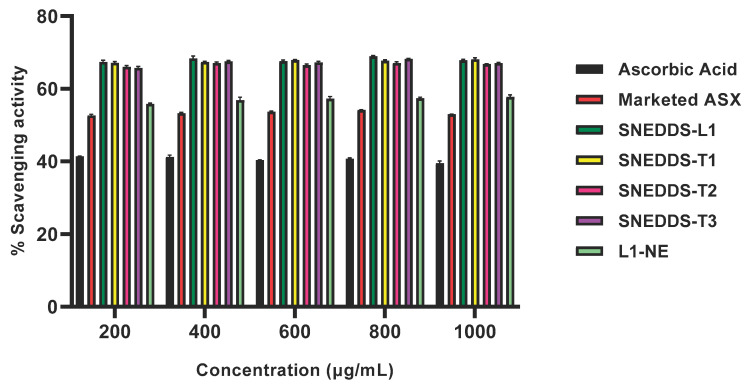
% ABTS-radical scavenging activity of 200–1000 µg/mL concentrations of SNEDDS formulations, SNEDDS-based nanoemulsion (L1-NE), marketed ASX topical product and ascorbic acid solution as positive control (mean ± SD; *n* = 3).

The ABTS radical scavenging assay was performed to investigate the antioxidant activity of all SNEDDS formulations and the SNEDDS-based nanoemulsion (L1-NE), and compared to the marketed topical product and ascorbic acid (Figure 5). The ABTS radical scavenging was stable across the concentration range of the formulations. At 1000 µg/mL, % scavenging activity in all SNEDDS was from 68.13 ± 0.44 µg/mL to 66.79 ± 0.08 µg/mL, and the L1-NE was approximately 20% lower at 57.79 ± 0.58 µg/mL. All SNEDDS-containing ASX had significantly greater ABTS scavenging activity than the ascorbic acid control (39.53 ± 0.61 µg/mL) and marketed topical product (53.00 ± 0.08 µg/mL; *p* < 0.05; Figure 5).

## 4. Discussion

The therapeutic potential of ASX has been investigated for a wide range of conditions and administration routes including oral and topical delivery [5]. Topical administration to the skin has been primarily for cosmeceutical purposes such as anti-aging with a number of facial serum products available. There is great potential to use the high antioxidant and anti-inflammatory capability of ASX for dermatological conditions such as repair of UV damage, dermatitis, and rashes. To fully utilise its potential, an effective delivery system is needed that targets ASX to the skin in therapeutic quantities. Targeted delivery of ASX to the epidermis and dermis, where it will exert its antioxidant and anti-inflammatory therapeutic activity, requires facilitating drug entry to these skin tissues whilst minimising uptake by the cutaneous circulation. The high lipophilicity of ASX is challenging as it requires formulating to enhance the solubility and delivery to the SC whilst also facilitating partitioning to the deeper more aqueous tissues of the epidermis and dermis. Previous reports have demonstrated the ability of self-emulsifying and self nano-emulsifying drug delivery systems (SEDDS and SNEDDS) to increase the solubility and bioavailability of lipophilic drugs including sesamin [32], clofazimine [38], meloxicam [34], and curcumin [48]. We proposed SNEDDS as a formulation approach to solubilize the highly lipophilic ASX and provide a delivery system that is simple to produce, has an attractive appearance, good spreadability on the skin, is stable (physically and chemically), and provides effective delivery of ASX into the skin to target its antioxidant/anti-inflammatory activity to its site of action in the epidermis and dermis. These criteria constituted the quality target product profile (QTPP) for assessing a successful formulation strategy [49,50]. We also proposed that the anhydrous SNEDDS formulations would perform better than a nanoemulsion composed from the same components as a SNEDDS formulation. Overall, in this study, the SNEDDS formulations containing ASX met the QTPP, with differences in the level of performance based on the choice of formulation excipients. All SNEDDS formulations performed better than the corresponding nanoemulsion, a marketed ASX facial serum product, and a solution of ASX in oil. 

SNEDDS formulations incorporating ASX composed of Labrafil^®^ M 1944 CS (constituting the oil phase), Kolliphor^®^ EL (non-ionic surfactant), and Transcutol^®^ P as a co-surfactant (designated SNEDDS-L1) were successfully formulated and provided statistically significant enhancement of skin permeation of ASX compared to a marketed ASX facial serum formulation and ASX in oil solution (Figure 3).

Labrafil^®^ M 1944 CS consists of mono-, di- and triglycerides and PEG-6 mono- and diesters of oleic (C18:1) acid. It is a nonionic water-dispersible surfactant for lipid-based formulations that is commonly used as a co-emulsifier in topical formulations, and provides the oil phase for the current SNEDDS formulations. The selected surfactant, Kolliphor^®^ EL (polyoxyl-35 hydrogenated castor oil), is a non-ionic solubilizer and emulsifying agent with hydrophile-lipophile balance (HLB) of 12. Its hydrophobic moiety is a combination of glycerol polyethylene glycol ricinoleate and fatty acid esters of polyethylene glycol, while the hydrophilic moiety is a combination of polyethylene glycols and glycerol ethoxylate [51]. Transcutol^®^ P, a high purity grade of diethylene glycol monoethyl ether (DEGEE), was selected as the co-surfactant because of its properties as a hydroalcoholic solubilizer [51] and skin permeation enhancer without compromising skin integrity [52,53]. All components are categorised as Generally Recognised as Safe and Effective (GRASE) by the U.S. Food and Drug Administration (FDA) [54]. 

The SNEDDS formulations provided excellent physical characteristics for skin application. The SNEDDS-L1 formulation showed small droplet size in the order of 18 nm, a negative zeta potential (12.40 ± 0.20 mV), and a viscosity (0.19 ± 0.01 Pas) that provided good spreadability and retention on the skin surface. Kaur and Ajitha reported that the delivery of fluvastatin increased when the droplet size and zeta potential are reduced and viscosity increased, suggesting that this was due to higher occlusivity on the skin [55]. The droplet size has also been shown to be an important parameter to enhance the release of the drug from the formulation [34,56]. Smaller droplet size resulted in a large surface area that provided high drug release of meloxicam from a SNEDDS for transdermal delivery [34]. In our preliminary studies, manufacturing without the high-energy ultrasonic homogenization step provided slightly larger droplets of approximately 26 nm. As this is still an acceptable size for skin delivery, it suggests that the SNEDDS formulations could be manufactured by a lower energy method that could be beneficial for industrial scale-up. The negative zeta potential (Table 2) increased electrostatic repulsion between droplets to enhance physical stability.

All ASX-containing SNEDDS formulations showed good physical stability over 30 days and significantly improved the chemical stability of ASX in the formulation compared to ASX in oil solution, particularly when exposed to light (Figure 4). Whilst this provides a preliminary indication of the stability of ASX in the SNEDDS formulations, a full stability study over a longer-term is required to assess the formulations for use as commercial products. As ASX confers its therapeutic effects via its antioxidant activity this was assessed in all SNEDDS formulations and a range of controls including ascorbic acid as the antioxidant standard. The SNEDDS formulations all demonstrated higher antioxidant activity than ASX in oil solution and an ascorbic acid standard (Figure 5), showing that the SNEDDS formulations provided ASX inactive form more readily than when ASX was dissolved in oil. The SNEDDS-based nanoemulsion showed lower antioxidant activity than its corresponding anhydrous SNEDDS formulation. This suggests that the ASX is more available from the SNEDDS formulation or that the ASX could have been partially degraded by the presence of dissolved oxygen in that water phase of the nanoemulsion, as has been suggested by Sun et al. [28]. In addition, it has been reported that ASX can self-assemble in polar solvents to form tightly packed stacks of individual molecules [57], potentially reducing its availability and activity.

The SNEDDS-L1 formulation enhanced the penetration of ASX in the skin, providing a 2.2-fold increase and a 3.6-fold increase in the SC and (E + D + F), respectively compared to marketed ASX product. As ASX is highly lipophilic, it is expected to penetrate the lipid bilayer regions of the SC, but have poor partitioning to the more aqueous deeper epidermal and dermal tissues that constitute the target site for the ASX antioxidant and anti-inflammatory activity. When applied as the commercial formulation, the deposition of ASX in the E + D + F was lowest, followed by the oil solution. The SNEDDS-L1 formulation significantly increased ASX penetration to the target tissues compared to both the marketed topical product and a solution of ASX in oil. This demonstrates that the SNEDDS formulation components and their nanosystem structure has the ability to enhance skin penetration by a number of mechanisms. First, the oil can solubilize the lipophilic ASX thus presenting it to the skin in a form that can access the lipid domains in the SC. However, that would not explain the significant improvement in comparison to the ASX in oil solution. Second, the oil, surfactant, and co-surfactant components can modify the SC lipid structure to facilitate permeation within the SC and entry to the viable epidermal regions. Third, presenting the ASX as a nanosystem optimizes ASX presentation to the skin, and facilitates diffusion and partitioning within the epidermal layers. Indeed, it has been postulated that SNEDDS formulations act by taking up moisture from the skin surface to form an occlusive topical system that has a high thermodynamic driving force for skin delivery [34,39,40]. We investigated formation of a SNEDDS-based nanoemulsion by addition of water to form L1-NE. However, when applied to the skin this nanoemulsion formulation showed significantly lower ASX penetration to the SC and (E + D + F) than the anhydrous SNEDDS-L1. This may be due to the increase in droplet size of the nanoemulsion compared to the SNEDDS-L1 (87.13 ± 2.44 and 18.79 ± 0.54 nm, respectively), or possibly a reduction in ASX degradation due to the presence of dissolved oxygen in the water phase. We did prepare all formulations immediately before administration in the IVPT studies so as to minimise any stability effects, but our antioxidant study did demonstrate a small reduction in ASX activity in the hydrous nanoemulsion compared to its corresponding anhydrous SNEDDS formulation (Figure 5).

Terpenes have demonstrated penetration enhancement activity by disruption of the intercellular lipid structure in the SC [58,59] to increase permeant diffusivity. Here, we investigated the addition of three terpenes into the oil phase to create three SNEDDS formulations: SNEDDS-T1 (D-limonene), SNEDDS-T2 (geraniol), and SNEDDS-T3 (farnesol). D-limonene (MW 1.36.23 g/mol; log Po/w 4.57) [60], geraniol (MW 154.25 g/mol; log Po/w 3.56) [61], farnesol (227.37 g/mol; log Po/w 5.77) [62] provide a range of chemical structures and properties that can influence their role in the formulation. 

Incorporation of terpenes increased ASX penetration to the deeper skin target site (E + D + F) compared to the SNEDDS-L1 formulation, with the following order of penetration SNEDDS-T1 > T2 > T3, although only the SNEDDS-T1 was significantly greater ASX penetration (*p* < 0.05: Figure 3). There was no significant difference in ASX penetration to the SC across the SNEDDS formulations, although again the order of ASX penetration was SNEDDS-T1 > T2 > T3. It is interesting to note that the presence of terpenes resulted in greater deep tissue penetration compared to SC deposition in all cases, suggesting that the terpenes are facilitating ASX diffusion within the skin tissues. Overall, the data suggest that inclusion of terpene does not increase release from the SNEDDS to the skin surface or penetration into the SC, but can improve partitioning from the SC into the more aqueous epidermal tissues and diffusion within the skin tissues. Previous studies have demonstrated a correlation between drug permeation from nanoemulsions with partition coefficient of the terpene in the formulation [63,64]. For example, El-Kattan et al. reported a positive correlation between the lipophilicity of the terpenes and the cumulative amount of hydrocortisone (log P 1.43) permeating through hairless mouse skin [63]. However, we did not see a similar correlation for the highly lipophilic ASX. El-Kattan et al. also examined the effect of terpene enhancers on solutes with a range of lipophilicities (−1 to 8 approximately) showing that terpenes-based skin permeation enhancement (range 135-fold to 2-fold) was inversely correlated with log P [65]. 

We have demonstrated that SNEDDS are an effective formulation for enhanced skin delivery of the highly lipophilic ASX to the deeper skin tissues that constitute the target site for therapeutic antioxidant and anti-inflammatory activity. We have also shown that they provide the ASX in active form with high antioxidant activity (Figure 5) and help to preserve stability of this active ingredient that is prone to degradation (Figure 4). As has been previously stated [38], the simplicity and ease of preparation of SEDDS and SNEDDS, compared to the manufacture of liposomes and nanoemulsions, provides advantages for topical/transdermal delivery systems of lipophilic molecules and has potential for poorly stable drugs and biologics.

## Figures and Tables

**Table 1:** ASX-SNEDDS formulation compositions (all excipients as *w*/*w*).

Codes	ASX	Labrafil^®^ M 1944 CS	Kolliphor^®^ EL	Transcutol	D-Limonene	Geraniol	Farnesol	Water
SNEDDS–L1	0.003	1.00	4.00	1.00				
SNEDDS–T1	0.003	0.95	4.00	1.00	0.05			
SNEDDS–T2	0.003	0.95	4.00	1.00		0.05		
SNEDDS–T3	0.003	0.95	4.00	1.00			0.05	
L1-NE	0.006	1.00	4.00	1.00				6.00

SNEDDS = self-nanoemulsifying drug delivery systems. L1 = initial formulation; T1 = addition D-limonene in oil phase; T2 = addition geraniol in oil phase; T3 = addition farnesol in oil phase. NE = nanoemulsion (50% water added to the oil/surfactant-cosurfactant mixture immediately before use). The final concentration of ASX in L1–NE was same concentration the SNEDDS.

**Table 2:** Physical characteristics of ASX self-nanoemulsifying formulations (mean ± SD; *n =* 3).

Codes	Droplet Size (nm)	PDI	Zeta Potential (mV)	Viscosity (Pas)	Refractive Index	pH	
						Before Dilution	After Dilution
SNEDDS–L1	18.79 ± 0.54	0.24 ± 0.02	−12.40 ± 0.20	0.19 ± 0.01	1.46 ± 0.01	8.01 ± 0.02	5.42 ± 0.01
SNEDDS–T1	18.44 ± 0.41	0.21 ± 0.00	−12.67 ± 0.21	0.19 ± 0.01	1.46 ± 0.01	8.01 ± 0.03	4.97 ± 0.04
SNEDDS–T2	18.95 ± 1.48	0.25 ± 0.02	−12.80 ± 0.35	0.20 ± 0.01	1.46 ± 0.01	7.97 ± 0.02	5.06 ± 0.03
SNEDDS–T3	17.75 ± 0.21	0.26 ± 0.02	−13.10 ± 0.52	0.23 ± 0.01	1.46 ± 0.01	7.89 ± 0.02	4.98 ± 0.08
L1-NE	87.13 ± 2.44	0.28 ± 0.00	−12.53 ± 0.55	0.22 ± 0.01	1.40 ± 0.01	6.17 ± 0.01	4.43 ± 0.01
Marketed ASX	160.00 ± 3.44	0.20 ± 0.00	−41.57 ± 2.46	0.01 ± 0.01	1.34 ± 0.01	5.40 ± 0.02 (no dilution)	

**Table 3:** Robustness to dilution studies of ASX-loaded SNEDDS (mean ± SD; *n =* 3).

Codes	Droplet Size (nm)		
	1:50	1:100	1:250
SNEDDS–L1	21.57 ± 0.05	18.79 ± 0.54	19.70 ± 0.27
SNEDDS–T1	19.30 ± 1.33	18.44 ± 0.41	18.47 ± 0.32
SNEDDS–T2	19.53 ± 1.35	18.95 ± 1.48	18.89 ± 0.50
SNEDDS–T3	18.08 ± 0.78	17.75 ± 0.21	19.03 ± 0.99
L1-NE	88.00 ± 3.71	87.13 ± 2.44	84.44 ± 4.52

**Table 4:** Skin distribution of ASX from SNEDDS formulations, marketed product, and oil solution control (mean ± SEM; *n* = 9).

Codes	ASX Distribution in the Skin (µg/cm^2^, mean ± SEM)		ER
	SC	E + D + F	
Control: ASX in oil	0.42 ± 0.01	0.56 ± 0.07	1.00
Marketed ASX product	0.67 ± 0.03	0.36 ± 0.03	1.05
SNEDDS–L1	1.48 ± 0.31	1.28 ± 0.29	2.82
SNEDDS–T1	1.40 ± 0.14	2.12 ± 0.19	3.59
SNEDDS–T2	1.09 ± 0.14	1.75 ± 0.10	2.90
SNEDDS–T3	0.82 ± 0.10	1.40 ± 0.06	2.27
L1–NE	0.50 ± 0.08	0.63 ± 0.24	1.15

ER = ratio of the mean total amount of ASX in skin (SC and E + D + F) from SNEDDS/ASX in oil solution. SC = stratum corneum; E + D + F = epidermis, dermis and follicles.

## Data Availability

Data available by email request.

## Supplementary Materials

The following are available online at https://www.mdpi.com/article/10.3390/pharmaceutics13050649/s1, Figure S1: HPLC chromatograms (peak area versus time) of: (A) 0.75 µg/mL ASX solution as prepared for the calibration curve; (B) SNEDDS-L1 formulation matrix without ASX; (C) SNEDDS-L1 formulation matrix with ASX incorporated; (D) Skin extract following administration of SNEDDS L1-NE in IVPT experiment.
