# Supplementary material for: Novel Self-Nano-Emulsifying Drug Delivery Systems Containing Astaxanthin for Topical Skin Delivery
Source: Pharmaceutics. 2021 May 3;13(5):649. doi: 10.3390/pharmaceutics13050649 (PMC8147608; doi:10.3390/pharmaceutics13050649)
Supplement: Supplementary file 1 [file pharmaceutics-13-00649-s001.zip › pharmaceutics-1148982-supp final.pdf]

pharmaceutics

Supplementary Materials: Novel Self-Nano-Emulsifying Drug Delivery Systems Containing Astaxanthin for Topical Skin Delivery

Thellie Ponto, Gemma Latter, Giuseppe Luna, Vânia R. Leite-Silva, Anthony Wright and Heather A. E. Benson

Methods

HPLC instrumentation and conditions

Chromatographic separation was performed using an Agilent™ 1200 system (Agilent Technologies, Waldbronn, Germany), equipped with a degasser, binary pump, autosampler, multiple wavelength detector (at 476 nm UV detection), and Chemstation Rev B.04.03-SP1 software. A Jupiter C18 5 µm column, (150 mm × 4.6 mm) protected by a Security Guard Cartridge (C18, 4 × 3 mm) both from Phenomenex (Lane Cove, NSW, Australia), was used with isocratic flow of mobile phase (methanol: water: dichloromethane (DCM) = 85:13:2) at a flow rate of 1 mL/min (chromatography adapted from Yuan *et al.* [1]. The autosampler temperature was under a controlled temperature of 10 °C with an illuminator setting.

HPLC analysis method development

The HPLC method was developed for analysis of ASX in pharmaceutical formulations and extraction of ASX from skin tissues.

Linearit*y*—Two series of ASX concentrations. System X (0.75–15 µg/mL) represented samples for determination of the solubility and stability of ASX, and the higher concentration tissue extract samples from skin permeation experiments with SNEDDS formulations. System Y (0.105–1.05 µg/mL) was for analysis of tissue sample extracts from commercial topical product administration in skin experiments.

Precision—ASX solutions at four concentrations (15, 3.75, 0.75 and 0.105 µg/mL) were analyzed in six replicates for each concentration. This provided concentrations from the two calibration curves used across the experimental samples. The acceptable relative standard deviation (RSD) < 5% [2].

Sensitivity—The blank solvent, a mixture of 15 mL of acetone and dichloromethane (50:50) and 10 mL mobile phase, was injected 6 times. The limit of detection (LOD) and LOQ (limit of quantification) were determined as three times and ten times the baseline noise level in the assay, respectively.

Accuracy—A mass balance study of ASX extracts from skin tissue samples that had been exposed to a SNEDDS formulation was carried out to assess the accuracy of the assay. The solvent extraction system was 50:50 ACT: DCM. The extraction procedure developed for the study involved soaking a pre-weighed section of skin tissue in 5 mL of an aqueous solution containing the SNEDDS-L1 formulation at a concentration of 0.0758 mg ASX/mL (equivalent to the theoretical concentration that would be present in an IVPT experiment) at 35 °C for 4h, then removing it, blotting dry and sectioning. The ASX was extracted from the skin sections (as per extraction protocol described in Section 2.6) and quantified by HPLC assay.

Results

HPLC assay method development

The HPLC method provided a single ASX peak for stock solution and good separation from any skin sample related peaks. ASX retention time was 8.3 ± 0.1 minutes with a

total analysis time of 13 minutes (Figure 1S). Two calibration curves were generated to allow for the larger volume injections in the lower concentration range calibration curve.

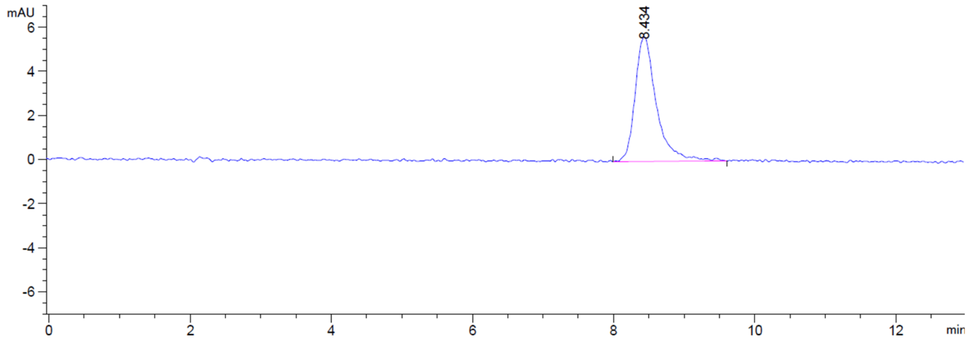

(A)

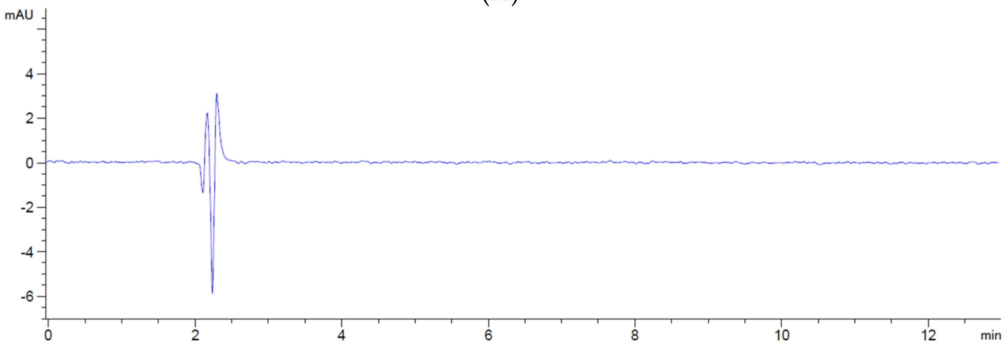

(B)

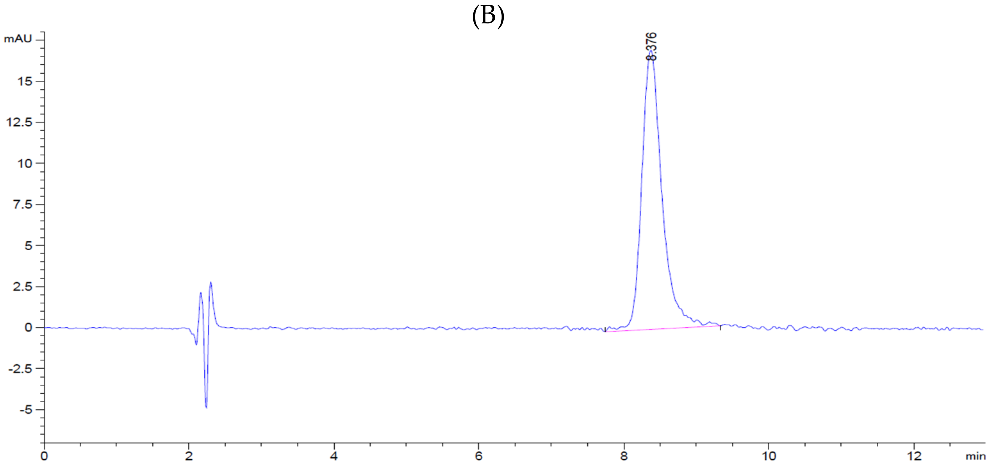

(C)

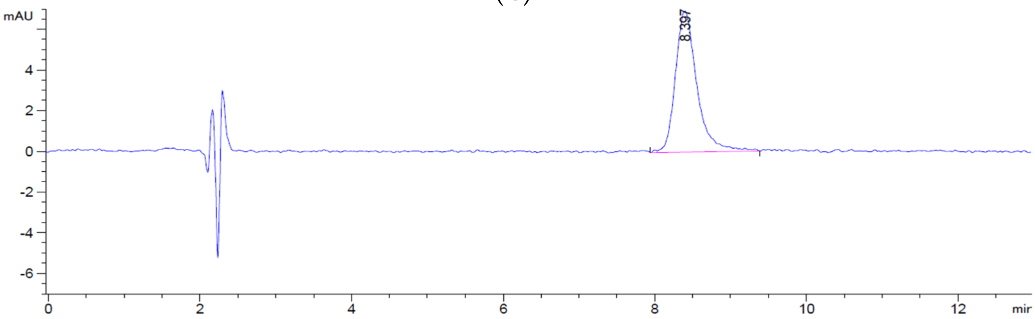

(D)

Figure S1. HPLC chromatograms (peak area versus time) of: (**A**) 0.75 µg/mL ASX solution as prepared for the calibration curve; (**B**) SNEDDS-L1 formulation matrix without ASX; (**C**) SNEDDS-L1 formulation matrix with ASX incorporated; (**D**) Skin extract following administration of SNEDDS L1-NE in IVPT experiment.

Linearity

The assay showed good linearity (regression coefficient of 0.99) for both concentration series.

Assay precision

The coefficient of variation precision parameters calculated from the relative standard deviation (n = 6) were 1.57% for 15 μg/mL, 4.18% for 0.105 μg/mL, 1.09% for 0.75 μg/mL, and 1.00% for 3.75 μg/mL. The acceptability criterion of precision repeatability has been fulfilled with R.S.D. < 5% in the high, medium, and low concentrations.

Sensitivity

The minimum LOD and LOQ for determination of ASX, calculated by injecting samples and blank solution six times were 41.7 ng/mL and 72.2 ng/mL respectively.

Accuracy

Figure S1 shows representative chromatograms for ASX analysis and control samples from the formulation development and IVPT experiments. This includes samples of the SNEDDS-L1 formulation without and with incorporation of ASX (B and C respectively), showing that there was no peak in the ASX region for the formulation matrix. The extraction fluid from skin that had been processed as per IVPT skin samples did not provide a peak in the ASX region (chromatogram not shown). The accuracy of the assay was determined from the recovery of ASX in the extraction from the skin permeation samples following administration of one SNEDDS formulation type. The amount of ASX recovered from the donor including washings, SC and skin extractions and receptor fluid samples was subtracted from the initial donor ASX applied gave a mass balance recovery of 88.87 ± 0.48% ASX compared to the amount of ASX applied.

Reference

1. Yuan, J.-P.; Chen, F. Chromatographic separation and purification of trans-astaxanthin from the extracts of Haematococcus pluvialis. *J. Agric. Food Chem.* **1998**, *46*, 3371–3375.
2. Namjoshi, S.; Caccetta, R.; Edwards, J.; Benson, H.A.E. Liquid chromatography assay for 5-aminolevulinic acid: application to in vitro assessment of skin penetration via Dermaportation. *J. Chromatogr. B Analyt. Technol. Biomed. Life Sci.* **2007**, *852*, 49–55.
